# Oral Fluid Testing for Pertussis, England and Wales, June 2007–August 2009 

**DOI:** 10.3201/eid2006.131069

**Published:** 2014-06

**Authors:** Helen Campbell, Gayatri Amirthalingam, Norman K. Fry, David Litt, Timothy G. Harrison, Karen Wagner, Natasha S. Crowcroft, Elizabeth Miller

**Affiliations:** Public Health England, Colindale, London, UK (H. Campbell, G. Amirthalingam, N.K. Fry, D. Litt, T.G. Harrison, K. Wagner, E. Miller);; Public Health Ontario, Toronto, Ontario, Canada (N.S. Crowcroft)

## Abstract

Existing pertussis surveillance systems tend to underidentify less severe cases among older children and adults. For routine follow-up of notified, nonconfirmed, clinically diagnosed pertussis cases, use of an oral fluid test was pilot tested in England and Wales during June 2007–August 2009. During that period, 1,852 cases of pertussis were confirmed by established laboratory methods and another 591 by oral fluid testing only. Although introduction of serologic testing in 2002 led to the greatest increase in ascertainment of pertussis, oral fluid testing increased laboratory ascertainment by 32% overall; maximal increase (124%) occurred among children 5–9 years of age. Patients whose pertussis was confirmed by oral fluid testing were least likely to be hospitalized, suggesting that milder community cases were being confirmed by this method. Oral fluid testing is an easily administered, noninvasive surveillance tool that could further our understanding of pertussis epidemiology and thereby contribute to decisions on vaccination strategies.

Existing surveillance systems for pertussis are incomplete and tend to be biased toward identifying severe cases in infants (*1*,*2*), as reflected by extremely high reported incidence for this age group (*3*–*5*). Underascertainment of cases in older patients is well recognized because of a combination of factors, including reduced likelihood that patients with milder symptoms will seek health care, underdiagnosis for patients who do seek health care, and underreporting (*6*–*9*). Although these persons usually experience milder disease, often without classic signs and symptoms, some become substantially ill (*9*) and can still infect vulnerable infants. Additional test methods that provide adequate sensitivity and specificity and that are acceptable to health professionals and patients could therefore improve ascertainment of pertussis and provide data that are more representative of disease within the population.

Since the early 1960s, *Bordetella pertussis* has been isolated from the nasopharynx by use of conventional microbiological techniques. However, culture requires that a specimen be collected early in the illness and might lack sensitivity because the organism is delicate and any delay in processing specimens can reduce the probability of isolation (*10*). Isolation of the organism is more difficult if the patient has been vaccinated, if the patient has received antimicrobial drugs, and if too much time has gone by since onset of cough. PCR testing for the presence of *B. pertussis* DNA in nasopharyngeal samples is more sensitive than culture because the organism does not need to be viable (*11*). PCR sensitivity, however, decreases substantially with increasing patient age and duration of symptoms (*12*). Serologic testing has been established as a diagnostic method complementary to PCR, and recommendations by European Union reference laboratories for such assays have been described (*13*). Serologic testing is used in at least 20 European countries (*14*) (including the United Kingdom since 2002), Japan, and Australia to diagnose infection in patients who have been coughing for at least 2 weeks, when culture and PCR are less likely to yield positive results (*10*) as has been demonstrated in certain studies (*15*). Serologic testing has been used predominantly for older children and adults who tend to seek care later (*5*).

The Health Protection Agency (HPA; which became Public Health England on April 1, 2013) Respiratory and Systemic Infection Laboratory (which became the Respiratory and Vaccine Preventable Reference Unit on April 1, 2013) developed an ELISA to detect IgG against pertussis toxin in oral fluid (*16*). This test was intended to act as a surrogate for the serum antibody assay. Oral fluid sampling is appealing because collection is straightforward; it is noninvasive (oral fluid is collected from around the gum line by using an absorbent swab) and can be collected by the patient or parent/guardian in the home and mailed to the laboratory for testing. The oral fluid assay detects seropositivity with a sensitivity of 79.7% (95% CI 68.3%–88.4%) and a specificity of 96.6% (95% CI 91.5%–99.1%) (*16*). Thus, oral fluid titers of >70 arbitrary units have a positive predictive value of 76.2%–93.2% for pertussis among children with chronic cough when used as a surrogate for the serum ELISA, assuming disease prevalence of 12%–37% (which includes the lower and upper limits of disease prevalence shown by other studies) (*17*).

Similar oral fluid antibody tests have been developed by HPA as surrogates for serologic testing for measles, mumps, and rubella (*18*,*19*). Oral fluid testing of patients after their formal notification of clinically diagnosed measles, mumps, and rubella has been conducted in England and Wales since 1994; this test has been acceptable and is used to augment routine serologic diagnosis for these diseases (*18*). After completion of a successful small-scale study in 2 areas of England (*20*), it was decided to conduct a national pilot test for the use of oral fluid testing for pertussis as a similar surveillance tool to obtain laboratory confirmation of pertussis cases statutorily notified on the basis of clinical diagnosis. Oral fluid testing was chosen because of the ease of sample collection and the predicted increased patient compliance with use of a noninvasive testing method. All laboratory-confirmed cases of pertussis are coordinated on a national basis, and each is followed up by asking the Health Protection Units (HPUs) or patients’ general practitioners to complete a detailed surveillance questionnaire.

The aim of this national oral fluid surveillance was to improve case ascertainment and representativeness, increase rates of confirmation of notified cases, and provide more detailed information on notified cases of pertussis (if confirmed) via the surveillance questionnaire. Such improvements would strengthen the evidence base for vaccination policy decision making. We compared the effects of oral fluid testing as a notification follow-up service over the 27 months that it was available with effects during a comparable 27 months before its availability.

## Methods

During June 2007–August 2009, a new oral fluid testing service was pilot tested throughout England and Wales. This testing service was provided free of charge by the HPA Respiratory and Systemic Infection Laboratory and Immunisation Department, through the 25 local HPA HPUs. The aim of this service was to seek laboratory confirmation of formally notified pertussis cases for which the patient had been coughing for at least 2 weeks but a diagnosis had not been confirmed by another available method (culture, PCR, or serologic testing).

By law, the Proper Officer of the local HPU should be notified of pertussis cases that are diagnosed by clinicians in hospitals or primary care settings within the geographic area of the HPU’s responsibility. When a case was reported, if the diagnosis had not been confirmed by culture, PCR, or serologic testing, the HPU mailed oral fluid sampling kits either directly to the patients or to the parents/guardians of patients (the kit was suitable for use at home) or to their general practitioner. The sampling kit contained an ORACOL saliva collection swab (Malvern Medical Developments Ltd, Worcester, UK), instructions, and a simple laboratory form for completion by patients. Detailed instructions were included in each kit, and they described how to collect the sample by brushing the swab along the gum line for 2 minutes.

Swabs were returned directly to the Respiratory and Systemic Infection Laboratory for testing in preaddressed packaging with prepaid postage, which was also included in the kit. Oral fluid was eluted from each swab and tested for IgG against pertussis toxin by ELISA as previously described (*16*). For patients who had been coughing for at least 2 weeks, a titer of >70 arbitrary units was considered consistent with recent infection in the absence of pertussis vaccination within the previous 12 months; as with serologic testing, antibodies from recent vaccination with pertussis vaccine can potentially confound test results used to provide markers of recent infection (*17*). Thus, a positive result for those who had received a pertussis-containing vaccine within 1 year before specimen collection cannot be easily interpreted. A surveillance form, identical to that used for all laboratory-confirmed cases, was sent to the local HPU for collection of additional information, including the patient’s vaccination history. We excluded completely from the dataset any patients whose oral fluid or serologic testing result was consistent with recent pertussis infection and who had received a pertussis-containing vaccine in the previous 12 months through this enhanced surveillance. 

If primary testing (PCR, serologic testing, and oral fluid testing) was undertaken, information for all samples submitted for testing, regardless of test result, was available for analysis. PCR, with real-time assay, was offered for hospitalized infants throughout the study period (*11*). Serologic testing for the detection of IgG against pertussis toxin based on single high-titer serologic results, considered indicative of recent infection (*21*,*22*), was offered for patients who had been coughing for at least 2 weeks. PCR and serologic testing were not routinely offered by other laboratories in England and Wales at the time of this study. Culture of *B. pertussis* from patient samples was undertaken in hospital diagnostic laboratories throughout England and Wales, and only positive results were reported to the Immunisation Department. These laboratories have been encouraged to submit putative *B. pertussis* isolates to the HPA Respiratory and Systemic Infection Laboratory for confirmation and national surveillance purposes. Therefore, data were available only for culture-confirmed (i.e., not culture-negative) cases, and a complete dataset of samples submitted for testing by culture in England and Wales was not available for analysis. For any given patient, >1 test sample might have been submitted. When data are shown by person, testing is presented in the following order: culture, PCR, serologic testing, oral fluid testing. Therefore, a patient with a positive culture and serologic testing result, for example, would be considered culture positive, and a patient would be considered oral fluid positive if this was the only positive test result.

In addition to analyzing laboratory-confirmed cases, we also analyzed pertussis notifications. Clinically notified cases were rendered anonymous and thus could not be linked to laboratory-confirmed cases for which full patient details were available. Routine follow-up (for epidemiologic data) of laboratory-confirmed cases of pertussis was in place throughout the study. Information about whether the patient was hospitalized was used to determine whether there was evidence that the profile of cases confirmed through oral fluid testing differed from that of cases confirmed through other established methods. By using logistic regression (Stata version 9, StataCorp, College Station, TX, USA), taking age and sex into account, we compared the risk for hospitalization (as an indicator of serious disease) by various test methods.

## Results

**Figure1:**
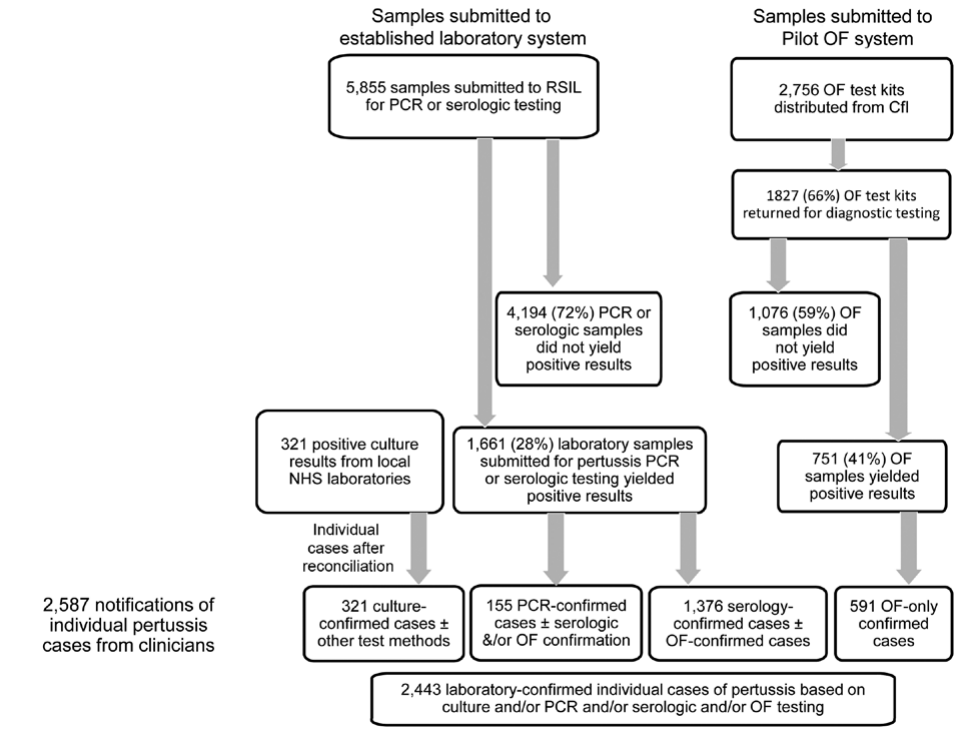
Distribution of notified cases of pertussis and pertussis samples submitted to the Health Protection Agency (HPA; became Public Health England on April 1, 2013) Respiratory and Systemic Infection Laboratory (RSIL; became the Respiratory and Vaccine Preventable Reference Unit on April 1, 2013) and collated by the HPA Immunisation Department, June 2007–August 2009, England and Wales. OF, oral fluid; NHS, National Health Service; CfI, Centre for Infections.

During the oral fluid pilot testing period, 2,756 oral fluid kits were sent to HPUs, 2,587 clinical cases were reported, and 2,443 cases of pertussis were confirmed by at least 1 laboratory method (Figure 1). Of these confirmed cases, 751 were confirmed by oral fluid testing plus or minus other methods and 591 (24% of all cases) were confirmed by oral fluid testing only, which increased laboratory ascertainment of pertussis by 32% (591 confirmed by oral fluid only/1,852 overall [confirmed by other methods ± oral fluid testing]), from 6% in <1 year to 124% in 5–9 years, assuming that these cases would not have been confirmed by other laboratory methods in the absence of oral fluid testing. During the pilot-testing period, 1,827 oral fluid samples were submitted and analyzed; thus, a high proportion (66%) of test kits distributed to HPUs resulted in samples being submitted for diagnostic testing.

During the pilot testing period, 29% (1,465) and 26% (196) of samples tested by serology and PCR, respectively, yielded positive results, and 41% (751) of the oral fluid samples yielded positive results (Table 1). The largest number of oral fluid samples was submitted from patients >20 years of age. However, the highest proportion with positive results (61%) were children 10–14 years of age; the availability of oral fluid testing mostly increased the total percentage of confirmed cases in children 1–9 years of age; during the pilot testing period, 52% and 55% of laboratory-confirmed cases in children 1–4 and 5–9 years of age, respectively, were confirmed by oral fluid testing alone, as were 36% of cases in children 10–14 years of age, compared with 21% for patients >15 years of age (Table 2). Taking 2008 as a year when oral fluid data had been available for a complete year, combining cases confirmed by oral fluid testing only with cases confirmed by the established methods resulted in an increase in disease incidence from 1.3 to 2.4 cases/100,000 children 1–4 years of age; from 0.8 to 2.1 cases/100,000 children 5–9 years of age; from 4.3 to 6.5 cases/100,000 children 10–14 years of age; and from 1.2 to 1.5 cases/100,000 persons >15 years of age.

**Table 1:** Distribution of samples received by RSIL for pertussis testing, England and Wales, June 2007–August 2009*

Patient/Age, y	Test method, no. submitted/no. positive (% positive)†				Notifications, no.
Oral fluid	PCR‡	Serology	Total
<1§	139/69 (50)	682/187 (27)	208/39 (19)	1,029/295 (29)	452
1–4	288/85 (30)	41/5 (12)	299/53 (18)	628/143 (23)	366
5–9	214/83 (39)	6/1 (17)	183/54 (30)	403/138 (34)	250
10–14	282/173 (61)	13/3 (23)	429/242 (56)	724/418 (58)	372
>15	904/341 (38)	19/0 (0)	3,975/1,077 (27)	4,898/1,418 (29)	1,147
All	1,827/751 (41)	761/196 (26)	5,094/1,465 (29)	7,682/2,412 (31)	2,587

*RSIL, Health Protection Agency (which became Public Health England on April 1, 2013) Respiratory and Systemic Infection Laboratory (which became the Respiratory and Vaccine Preventable Reference Unit on April 1, 2013). Data are based on samples submitted, and >1 sample/patient might have been submitted. †Culture testing is excluded because RSIL does not undertake initial culture testing; therefore, the total number of samples submitted for testing nationally is not known. ‡PCR testing is not routinely available for patients >1 y of age with suspected pertussis.  §Culture testing accounted for >50% of all pertussis confirmations for infants <1 y of age, but the total number of samples submitted for testing is not known.

**Table 2:** Total and proportion of confirmed pertussis cases, England and Wales, June 2007–August 2009

Patient age	Test method, no. (%)*				Total
	Culture	PCR	Serology	Oral fluid	
<3 mo	211 (60)	118 (34)	11 (3)	11 (3)	351
3–5 mo	46 (53)	27 (31)	4 (5)	10 (11)	87
6–11 mo	9 (53)	2 (12)	3 (18)	3 (18)	17
1–4 y	17 (14)	5 (4)	36 (30)	63 (52)	121
5–9 y	5 (4)	0	57 (41)	77 (55)	139
10–14 y	12 (3)	3 (1)	253 (60)	153 (36)	421
>15 y	21 (2)	0	1,012 (77)	274 (21)	1,307
All	321	155	1,376	591	2,443

*When >1 test method was used, culture takes precedence over PCR, which takes precedence over serology, which takes precedence over oral fluid testing (e.g., a case confirmed by culture and serologic testing is listed under culture).

**Figure 2:**
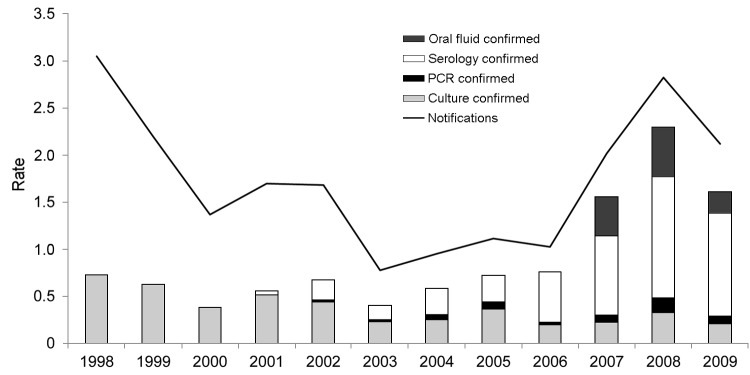
Rates of pertussis notification and laboratory confirmation (no. cases/100,000 population), by test method, England and Wales, 1998–2009. When >1 test method was used, culture takes precedence over PCR, which takes precedence over serology, which takes precedence over oral fluid (e.g., a case confirmed by culture and serologic testing is listed under culture).

When the distribution of confirmed cases by test method was considered over a longer period, which encompassed the introduction of routine serologic testing by the Respiratory and Systemic Infection Laboratory in January 2002, it was clear that the introduction of serologic testing had the greatest overall effect on testing for and ascertainment of pertussis in England and Wales, and the discrepancy between notified and laboratory-confirmed cases was correspondingly reduced (Figure 2). The proportion of cases confirmed by serologic testing increased with increasing patient age (Table 2); 77% (1,012/1,307) of all cases in patients >15 years of age were confirmed by serologic testing during June 2007–August 2009, and 18% of cases were confirmed for those 6–11 months of age. A higher proportion of positive results among patients >1 year of age were obtained by oral fluid testing (40%) than by serologic testing (29%). When the 79.7% sensitivity of oral fluid versus serologic testing is corrected for, ≈50% of the submitted oral fluid swab samples represented true cases of pertussis.

**Figure 3:**
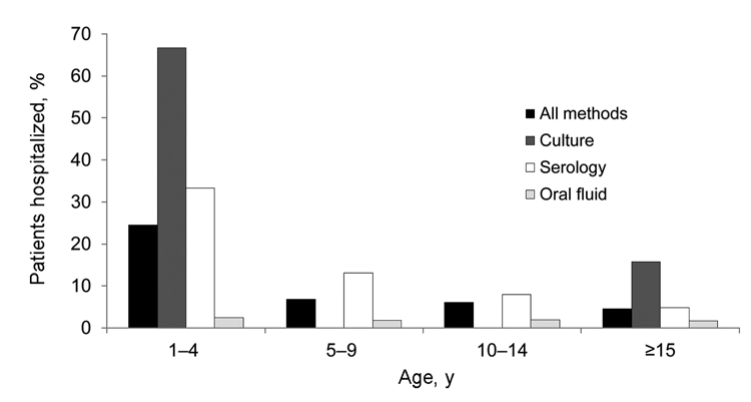
Proportion of cases hospitalized by age group and test method, England and Wales, June 2007–August 2009. When >1 test method was used, culture takes precedence over PCR, which takes precedence over serology, which takes precedence over oral fluid (e.g., a case confirmed by culture and serologic testing is listed under culture).

Patients with oral fluid–confirmed pertussis were least likely to be hospitalized in each age group >1 year of age (Figure 3). Overall, among those >1 year of age (among whom hospitalization was less frequent than among infants and oral fluid testing was more widely used), patients tested by serology and culture were 6 (95% CI 2.6–13.8) and 15 (95% CI 5.2–44.9) times more likely to be hospitalized than were those tested by oral fluid, when age and sex were taken into account. This finding suggests that milder cases of pertussis in the community were being confirmed through oral fluid surveillance.

Total numbers of samples submitted for PCR, serologic testing, and oral fluid testing were compared with notifications during the same period (Table 1). More patients had samples submitted for testing than were formally notified at all ages, indicating substantial underreporting through the notification system despite the apparent improvement (Figure 2); notifications accounted for 56% of the number submitted for testing among those 1–14 years of age and 23% of those >15 years of age.

## Discussion

The aim of this study was to improve case ascertainment and representativeness, increase rates of laboratory confirmation of notified cases, and provide more detailed information about confirmed cases of pertussis by surveillance questionnaire. Among oral fluid samples submitted during the pilot testing period, 751 (41%) had positive results, compared with 1,465 (29%) and 196 (26%) samples tested by serology and PCR, respectively. The highest proportion of positive samples (61%) was from children 10–14 years of age, and the availability of oral fluid testing most increased the total number of confirmed cases among children 1–9 years of age.

In the United Kingdom, pertussis vaccine is offered for infants at 2, 3, and 4 months of age; a booster dose 3 years after completion of the primary course is also offered. Since 1992, vaccine coverage by a child’s first birthday has been >90%, and since 2009–2010, receipt of the booster dose has been >85%. The primary aim of the pertussis immunization program is to minimize disease, hospitalization, and death among young infants. Despite these sustained high levels of coverage, increased pertussis activity occurred in England and Wales starting in October 2011, leading to declaration of a national outbreak in April 2012 (*23*,*24*). During this outbreak, the highest reported incidence of disease was among infants <6 months of age, followed by adolescents 10–14 years of age. In response to the continued increases in disease levels observed among young infants, the UK Departments of Health introduced a temporary program to offer pertussis vaccination to pregnant women; the program started in October 2012 and continued while disease levels remained high (*25*). This program passively protects infants from birth, through intrauterine transfer of maternal antibodies, until they could be actively protected by the routine infant vaccination program.

Over recent years, several other countries, including the United States (*26*), Australia (*27*), and Canada (*28*,*29*), have experienced increased pertussis activity, and these 3 countries have made PCR testing widely available. However, the availability of a noninvasive test method for children might be more acceptable to parents/guardians and health professionals, especially among children who are not severely ill. The lower proportion of samples submitted for serologic pertussis testing for children 1–14 years of age (compared with the percentage submitted for adults) suggests that providing blood samples is unpopular and that oral fluid is a useful alternative. Milder illness is also more likely to result in persons seeking care later in the course of illness, when culture and PCR are less sensitive. Similarly, the data collected through national pilot testing in England and Wales suggest that oral fluid surveillance improved ascertainment of milder cases beyond those confirmed through the testing that was already in place (culture, PCR [infants only], and serology). This improved ascertainment is useful because mild cases are problematic for surveillance because they are underdiagnosed and contribute to sustained transmission of pertussis within the community. Furthermore, underascertainment of milder infections causes bias, leading to overestimation of vaccine effectiveness (*30*). Unlike other available methods, oral fluid testing was acceptable for self-sampling and did not require health care provider time or expertise, which made it more cost-effective for surveillance. Oral fluid testing has been considered ideal for the primary care setting (*31*).

Successful programs for vaccination of pregnant women would directly reduce the number of cases among infants <3 months of age. If high levels of activity persist in other age groups, however, increased risk of acquiring infection during infancy would also persist. The finding that the level of positivity for samples submitted for diagnostic testing is low underlines how problematic diagnosis of pertussis can be for patients in age groups that tend to not show classic symptoms. Higher rates of positivity among adolescent/teenage children may be consistent with a real increased risk for pertussis for persons in this age group or could indicate continued underascertainment of cases in this age group despite the availability of a noninvasive test method.

In view of the increased disease incidence among children 10–14 years of age that started in October 2011 and the concerns that serologic testing might be suboptimal for patients in this age group, in 2013, oral fluid testing was made routinely available in England for patients 5–16 years of age with cases of pertussis that had not been confirmed by other laboratory methods (*32*). Making noninvasive testing available for patients in this age group is considered prudent, given the increased number of cases observed among children 7–10 years of age and adolescents in other countries (*26*,*33*–*35*). There is ongoing discussion about the need for boosters for adolescents and the optimal age at which they should be administered. If immunity does wane more rapidly after vaccination with acellular pertussis vaccines than with whole-cell vaccines, then countries such as the United States and Canada might benefit from improved surveillance to further inform the timing of booster vaccinations.

Although residual antibodies from vaccinations received while in preschool could potentially affect some positive oral fluid results for children <8 years of age in this study (*17*), we excluded those known to be vaccinated <1 year before oral fluid (and/or serologic) testing only through routine follow-up. The individual titers of 14 patients with cases confirmed by oral fluid testing only at >1 and <3 years after booster pertussis vaccination were well above the cutoff with a high positive predictive value for each case (mean 96.8%, range 85.1%–100%), and on this basis it was highly likely that only true cases of pertussis were included.

In conclusion, the introduction of serologic testing followed by oral fluid testing has successively narrowed the gap in surveillance for pertussis in England and Wales. Broader use of PCR testing is currently being pilot tested in participating regions in England as a way to further improve pertussis surveillance for patients seeking care earlier in the course of illness. In countries that already widely use PCR and/or serologic testing, oral fluid testing could improve diagnosis for patients who seek care later in the course of illness, thereby ruling out other potential causes and preventing unnecessary intervention. Although the oral fluid assay is only performed at the Respiratory and Systemic Infection Laboratory, this technology has the potential for broader application and wider availability. Oral fluid testing is an additional surveillance tool that offers higher acceptability and lower cost than other available methods. 

Pertussis is a rapidly reemerging disease; in several countries, reported incidence rates are high already, and rates could yet increase in other countries where disease is currently well controlled. More complete ascertainment is needed globally to better understand pertussis epidemiology and transmission, thereby facilitating the development of improved vaccines and vaccination strategies to improve future disease control.
